# Amniotic Fluid Cathelicidin in PPROM Pregnancies: From Proteomic Discovery to Assessing Its Potential in Inflammatory Complications Diagnosis

**DOI:** 10.1371/journal.pone.0041164

**Published:** 2012-07-18

**Authors:** Vojtech Tambor, Marian Kacerovsky, Ctirad Andrys, Ivana Musilova, Helena Hornychova, Lenka Pliskova, Marek Link, Jiri Stulik, Juraj Lenco

**Affiliations:** 1 Biomedical Research Center, University Hospital Hradec Kralove, Hradec Kralove, Czech Republic; 2 Department of Obstetrics and Gynecology, Faculty of Medicine Hradec Kralove, Charles University in Prague, University Hospital Hradec Kralove, Hradec Kralove, Czech Republic; 3 Department of Clinical Immunology and Allergy, Faculty of Medicine Hradec Kralove, Charles University in Prague, University Hospital Hradec Kralove, Hradec Kralove, Czech Republic; 4 Department of Obstetrics and Gynecology, Faculty of Health Studies, Pardubice University, Hospital Pardubice, Pardubice, Czech Republic; 5 Fingerland’s Department of Pathology, Faculty of Medicine Hradec Kralove, Charles University in Prague, University Hospital Hradec Kralove, Hradec Kralove, Czech Republic; 6 Institute of Clinical Biochemistry and Diagnostics, Faculty of Medicine Hradec Králové, Charles University in Prague, University Hospital Hradec Králové, Hradec Kralove, Czech Republic; 7 Institute of Molecular Pathology, Faculty of Military Health Sciences, University of Defence, Hradec Kralove, Czech Republic; Lund University Hospital, Sweden

## Abstract

**Background:**

Preterm prelabor rupture of membranes (PPROM) complicated by microbial invasion of the amniotic cavity (MIAC) leading to histological chorioamnionitis (HCA) significantly impacts perinatal morbidity. Unfortunately, no well-established tool for identifying PPROM patients threatened by these disorders is available.

**Methodology/Principal Findings:**

We performed an unbiased exploratory analysis of amniotic fluid proteome changes due to MIAC and HCA. From among the top five proteins that showed the most profound and significant change, we sought to confirm results concerning cathelicidin (P49913, CAMP_HUMAN), since an ELISA kit was readily available for this protein. In our exploratory proteomic study, cathelicidin showed a ∼6-fold higher concentration in PPROM patients with confirmed MIAC and HCA. We verified significantly higher levels of cathelicidin in exploratory samples (women without both MIAC and HCA: median 1.4 ng/ml; women with both conditions confirmed: median 3.6 ng/ml; *p* = 0.0003). A prospective replication cohort was used for independent validation and for assessment of cathelicidin potential to stratify women with MIAC leading to HCA from women in whom at least one of these conditions was ruled out. We confirmed the association of higher amniotic fluid cathelicidin levels with MIAC leading to HCA (the presence of both MIAC and HCA: median 3.1 ng/ml; other women: median 1.4 ng/ml; *p*<0.0001). A cathelicidin concentration of 4.0 ng/ml was found to be the best cut-off point for identifying PPROM women with both MIAC and HCA. When tested on the validation cohort, a sensitivity of 48%, a specificity of 90%, a likelihood ratio of 5.0, and an area under receiver-operating characteristic curve of 71% were achieved for identification of women with MIAC leading to HCA.

**Conclusions:**

Our multi-stage study suggests cathelicidin as a candidate marker that should be considered for a panel of amniotic fluid proteins permitting identification of PPROM women with MIAC leading to HCA.

## Introduction

Preterm prelabor rupture of membranes (PPROM) occurs in one third of all preterm deliveries and represents a specific subset of spontaneous preterm deliveries. It is defined as spontaneous rupture of the membranes with the leakage of amniotic fluid at least two hours before the onset of regular uterine activity in the gestational age below 37 weeks [1].

Several areas of controversy in the management of PPROM pregnancies exist, but at least three of the most important strategies are widely accepted by the broad obstetrician community: i) the use of antibiotics to prolong the time period between rupture of the membranes and delivery, ii) the administration of corticosteroids below gestational age of 32 weeks to diminish the risk of respiratory disease in newborns, and iii) the application of magnesium sulfate for fetal neuroprotection [2]–[8]. Subsequently, either expectant or active management must be chosen. There is a little maternal benefit in expectant management, but there can be significant neonatal benefit from the prolongation of the pregnancy, which leads to the reduction of the gestational age–dependent morbidity [9], [10].

Microbial invasion of the amniotic cavity (MIAC), which complicates PPROM in approximately 30% of cases, may induce intraamniotic inflammatory response [11], [12]. Specific motifs on the bacterial surface as well as endogenous molecules, released from damaged tissue and cells, are recognized by pattern recognition receptors. Their activation leads to increasing levels of inflammatory mediators in the amniotic fluid followed by the recruitment of neutrophils and other immune cells from the uterine wall to the placenta and fetal membranes. The neutrophil infiltration of the placenta and fetal membranes is then called histological chorioamnionitis (HCA).

The parallel presence of both MIAC and HCA, determining an infectious phenotype of PPROM, is responsible for serious neonatal morbidity, including chronic pulmonary diseases [13], [14] and adverse neurodevelopmental outcome [15], [16], both of which have long-term consequences on quality of life and health care costs [1]. This suggests that the identification of the infectious phenotype of PPROM is crucial for improving outcome in expectant management and parental counseling of women at risk [17]. Regrettably, there is no robust diagnostic tool currently available for identifying this phenotype.

Aside from the hypothesis-based research approach widely used in the quest for new diagnostic biomarkers, proteomics offers an unbiased alternative view on the protein changes associated with diseases [18]. The possibility of identifying hundreds of proteins combined with the ability to quantify changes in their abundance across multiple samples makes proteomics a very appealing approach for the exploratory phase of the biomarker discovery process. Promising candidate proteins, selected upon completing this phase, should then be targeted using complementary methods to verify the initial findings and to further validate their diagnostic potential in larger independent patient cohorts [18].

Two pieces of pioneering work, by Gravett et al. and Romero et al., presented the potential of proteomics in the discovery of novel biomarkers of intraamniotic infection in spontaneous preterm birth patients [19], [20]. Unfortunately, the validation step of the exploratory proteomic phase findings is frequently neglected. Without these data, the translation of these candidate markers into subsequent preclinical and ultimately clinical trials is substantially limited. In line with the potential of proteomics in discovering new biomarker candidates, we sought to perform an exploratory multidimensional shotgun proteomic analysis to compare the proteome composition of amniotic fluid from PPROM women with confirmed MIAC and HCA against samples from PPROM women for whom these conditions were ruled out, with the ultimate goal of identifying novel potential markers of MIAC leading to HCA. Keeping in mind the need for proper validation of the findings, we further aimed to support our primary results by performing two-stage confirmation experiments with the most prospective prominent marker candidate. To maintain the unbiased nature of our approach, the most prospective candidate protein was chosen according to the degree of dysregulation and statistical significance of the change, with regard to the availability of a suitable commercial ELISA assay for confirmation.

## Methods

### Ethics Statement

This study was approved by the Institutional Review Board committee – Ethics Committee, University Hospital Hradec Kralove (March 19, 2008; No. 200804 SO1P). Written informed consent was obtained from all participants. Amniocentesis is routinely offered for the assessment of the microbial status of the amniotic cavity to all women who are admitted with diagnosis of PPROM to the Department of Obstetrics and Gynecology, University Hospital Hradec Kralove.

### Diagnosis of PPROM

PPROM was defined as the leakage of amniotic fluid prior to the onset of labor (by at least two hours). This condition was diagnosed visually using a sterile speculum examination to confirm pooling of amniotic fluid in the vagina together with a positive test for the presence of insulin-like growth factor–binding protein (ACTIM PROM test; MedixBiochemica, Kauniainen, Finland) in the vaginal fluid.

### Management of PPROM

Management of PPROM in the Czech Republic is active (except for pregnancies below 28 weeks of gestation) and occurs no later than 72 hours after the rupture of membranes [21].Induction of labor is initiated or elective cesarean section is performed depending on the gestational age (within 24 hours for gestational ages higher than 34 weeks, within 48 hours for gestational ages between 32 and 34 weeks, and within 72 hours for gestational ages between 28 and 31 weeks), the fetal status, maternal serum C-reactive protein concentrations, and cervicovaginal group B Streptococcus colonization.

### Amniotic Fluid Sampling

Ultrasound-guided transabdominal amniocentesis was performed upon admission prior to the administration of corticosteroids, antibiotics, or tocolytics; approximately 5 ml of amniotic fluid was aspirated. The amniotic fluid sample was divided into three polypropylene tubes (TPP, Trasadingen, Switzerland) and was processed immediately. Two tubes were transported to the laboratory for detection of genital mycoplasmas (*Ureaplasma spp.* and *Mycoplasma hominis*) and *Chlamydia trachomatis* using PCR analyses, and for aerobic and anaerobic cultivation. The third tube, designated for proteomic analyses, was supplemented with protease inhibitors (40 µL *per* 1 ml of sample; Complete Mini, EDTA-free Protease Inhibitor Cocktail; Roche Diagnostics, Basel, Switzerland), centrifuged for 15 minutes at 300×*g*, and filtered using a syringe-driven 0.22 µm filter (TPP) to remove cells and debris. The sample was divided into aliquots, which were stored at −80°C until analysis.

### Diagnosis of MIAC

MIAC was determined based on a positive PCR detection of genital mycoplasmas (*Ureaplasma spp., Mycoplasma hominis*) and/or the observation of the growth of any bacteria except for coagulase-negative *Staphylococcus epidermidis*, which was considered to be a skin contaminant.

### Diagnosis of HCA

The degree of neutrophil and leukocyte infiltration was assessed separately in free fetal membranes (amnion and chorion-decidua), in the chorionic plate, and in the umbilical cord according to the criteria given by Salafia [22]. HCA diagnoses were determined based on the presence of neutrophil infiltration in the chorion-decidua (grade 3–4), and/or the chorionic plate (grade 3–4), and/or the umbilical cord (grade 1–4), and/or the amnion (grade 1–4). Funisitis was diagnosed by the presence of neutrophil infiltration within the umbilical cord. A single perinatal pathologist who was blinded to the clinical status of the women reviewed all the histopathological samples.

### Sample Collection for Exploratory Cohort

A total of 72 women with PPROM were recruited. Histopathological assessment of the placenta and PCR analyses for genital mycoplasmas along with aerobic and anaerobic cultivation of the amniotic fluid were available for 66 women. The women were divided into groups according to the presence or the absence of MIAC and HCA. Women with the presence of MIAC and the absence of HCA (n = 4) and women with the absence of MIAC and the presence of HCA (n = 18) were excluded from the exploratory cohort. All women with both MIAC and HCA (n = 19) and those women without both MIAC and HCA (n = 19) having the same range of gestational age at sampling as found in the previous group (24+0–34+5) were included in the exploratory cohort. Therefore, six women without both MIAC and HCA having gestational age at sampling ≥34+6 were excluded. All women were recruited at the Department of Obstetrics and Gynecology, University Hospital Hradec Kralove, Czech Republic, between May 2008 and August 2009.

Gestational ages were established for all pregnancies based on the first trimester ultrasound evaluation. Women with the following criteria were enrolled into this cohort: singleton pregnancy, PPROM between the gestational ages of 24 and 36 weeks, maternal age above 18 years, and no ultrasound signs of intrauterine growth restriction. The exclusion criteria were structural malformations or chromosomal abnormalities of the fetus, maternal pre-pregnancy and pregnancy complications (hypertension, pre-eclampsia, diabetes mellitus, and thyroid disease), and signs of fetal hypoxia.

### Sample Collection for Replication Cohort

The sample size was calculated based on the verification results from the exploratory cohort. Based on the outputs, we created a replication cohort containing 40 women with MIAC and HCA as a minimum of 38 women was needed to reach 80% power with significance level 0.01. A prospective cohort study was performed. Pregnant women with gestational age between 24+0 and 36+6 weeks, who were admitted to the Department of Obstetrics and Gynecology, University Hospital Hradec Kralove, between September 2009 and August 2011 with diagnosis of PPROM were involved. In total, 113 women with PPROM were recruited. Results regarding histopathological assessment of the placenta and PCR analysis for genital mycoplasmas were not available for 8 (7%) and 2 (2%) women, respectively. Therefore, the remaining 103 women were included in the replication cohort. In all pregnancies, the gestational age was established based on first trimester ultrasound evaluation.

Women who met the following criteria were eligible for enrollment in the replication cohort: a singleton pregnancy with PPROM, maternal age higher than 18 years, no signs of small for gestational age (estimated fetal weight by ultrasound below 10^th^ percentiles for gestational age), no fetal structural malformations or chromosomal abnormalities, no maternal complications (hypertension, preeclampsia, diabetes mellitus, and thyroid disease), and no other medical complications during pregnancies. Exclusion criteria were vaginal bleeding or signs of fetal hypoxia. Altogether, 40 women confirmed to have both MIAC and HCA were compared against 63 women in whom at least one of these conditions was ruled out.

### Proteomics Exploratory Phase

#### Generation of pooled samples

Protein concentration was determined using the bicinchoninic acid assay kit (Pierce, Rockford, IL) in each of 38 exploratory cohort samples. An equal amount of protein was taken from each sample to create a pooled MIAC- and HCA-positive and a pooled negative sample, both in duplicate (Fig. S1). Each pooled sample contained 2 mg of protein. The volume was adjusted to 4 ml using the Multiple Affinity Removal System (MARS) buffer A (Agilent, Palo Alto, CA), and samples were concentrated using Amicon Ultra filters (Millipore, Bedford, MA) with a 3 kDa molecular weight cut-off. The retenates were collected and adjusted to 200 µl with MARS buffer A.

#### Immunoaffinity depletion of high-abundance amniotic fluid proteins

The 14 abundance proteins (albumin, IgG, antitrypsin, IgA, transferrin, haptoglobin, fibrinogen, alpha-2-macroglobulin, alpha-1-acid glycoprotein, IgM, apolipoprotein Al, apolipoprotein All, complement C3, and transthyretin) were removed using the MARS Hu-14 column (Agilent) on an Alliance 2695 HPLC system (Waters, Milford, MA) according to the manufacturer’s instruction. The flow-through fraction was collected between 5^th^ and 22^nd^ minute of separation. MARS buffer was exchanged three times for water using the 3 kDa cut-off Amicon Ultra filters. The retenates were collected, and total protein concentration was determined by the bicinchoninic acid assay.

#### Trypsin digestion

From each replicate, 200 µg of protein was brought to 40 µl of 250 mM triethylammonium bicarbonate buffer, pH 8.5 (Sigma, St. Louis, MO) containing 0.1% RapiGest (Waters). Proteins were reduced using 5 mM tris(2-carboxyethyl) phosphine hydrochloride (Sigma) for 1 h at 60°C and digested overnight at 37°C by trypsin (Promega, Madison, WI) at a 1∶50 trypsin-to-protein ratio.

#### Application of CysTRAQ

The CysTRAQ technology was applied according to our recently published method [23]. Briefly, peptides were labeled with iTRAQ (isobaric tags for relative and absolute quantitation) reagents (AB Sciex, Foster City, CA) for 2 h. The MIAC- and HCA-negative pooled samples were labeled with 114 and 116 tags, while MIAC- and HCA-positive pooled samples were labeled with 115 and 117 tags. After the labeling step, the samples were diluted three-fold with water, combined, acidified using TFA (Sigma) to pH 1–2 and incubated for 30 min at 37°C to hydrolyze both RapiGest as well as unreacted iTRAQ tags. The hydrophobic part of RapiGest was removed by centrifugation and the supernatant was desalted using an Oasis HLB 1cc (30 mg) SPE column (Waters), and vacuum dried. Twenty-five milligrams of the Thiopropyl-Sepharose 6B thiol affinity resin powder (GE Healthcare, Uppsala, Sweden) was rehydrated in water and washed with coupling buffer (50 mM Tris, 1 mM EDTA, pH 7.5). Peptides were dissolved in 20 µl of 5 mM dithiothreitol (Sigma) in coupling buffer and reduced for 1 h at 60°C. The sample was diluted to 100 µl with coupling buffer and incubated with the slurry for 2 h at 37°C. Unbound non-cysteinyl peptides were captured using the Macro SpinColumn (Harvard Apparatus, Holliston, MA). The beads were then washed with 2.5 ml of each of the following solutions: washing buffer (50 mM Tris, 1 mM EDTA, pH 8.0); 2 M NaCl; 80% acetonitrile (ACN), 0.1% TFA; and washing buffer. Bound peptides (cysteinyl peptides) were released by incubation with 100 µl of 50 mM dithiothreitol in washing buffer for 1 h at 60°C. Both fractions were desalted on Oasis HLB 1cc (10 mg) Extraction Cartridge SPE columns and dried. The peptides were reduced with 5 mM tris(2-carboxyethyl) phosphine hydrochloride for 1 hour at 60°C, and cysteines were blocked with 10 mM methyl methanethiosulfonate (AB Sciex) for 10 min at RT.

#### Basic pH reversed-phase peptide fractionation

Desalted cysteinyl and non-cysteinyl peptide fractions were redissolved in 200 µl of 20 mM ammonium formate (NH_4_FA). The fractionation was performed on the Alliance 2695 HPLC system. Non-cysteinyl peptides (100 µl) and cysteinyl peptides (200 µl) were injected onto a Gemini C_18_ 150×2 mm column (Phenomenex, Torrance, CA) filled with 3 µm, 110 Å particles. The peptides were separated by a linear gradient, from 5% ACN, 20 mM NH_4_FA to 55% ACN, 20 mM NH_4_FA in 62 min. The eluting peptides were collected between 20 and 60 min of separation resulting in 18 collected fractions *per* sample. Each fraction was acidified with formic acid, and the samples were dried *in vacuo*.

#### LC-MS/MS analysis

Each basic pH fraction was redissolved in 40 µl of 5% ACN, 0.1% TFA, following nanoLC peptide separation on an UltiMate3000 HPLC system (Dionex, Sunnyvale, CA). Peptides were desalted on a µ-Precolumn 300 µm×5 mm filled with C_18_PepMap, 5 µm, 100 Å particles (Dionex). The peptides were separated on an analytical NanoEase column 100 µm×150 mm filled with Atlantis C_18_, 3 µm, 100 Å particles (Waters) by a linear gradient starting at 5% ACN, 0.1% TFA, and going to 50% of 80% ACN, 0.1% TFA in 85 min at a flow rate of 360 nl/min. The Probot fraction collector (Dionex) collected fractions every 8 s for 60 min onto an OptiTOF LC-MALDI plate (AB Sciex). The eluate was mixed 1∶4 post-column with 3 mg/ml α-cyano-4-hydroxycinnamic acid matrix (LaserBio Labs, Sophia-Antipolis, France) in 70% ACN, 0.1% TFA. The MALDI analysis was performed on a 4800 MALDI-TOF/TOF Analyzer (AB Sciex). MS spectra were acquired across the mass range of 800–4000 *m/z* using 625 laser shots *per* spectrum. A maximum of 12 precursors were chosen for fragmentation in each MS spectrum, starting with the weakest precursor. Collision-induced dissociation MS/MS spectra were acquired with a total accumulation of 3000 laser shots.

#### Data analysis

Spectra evaluation was conducted in ProteinPilot 2.0.1 software (AB SCIEX) using the Paragon search algorithm, Pro Group algorithm, and the integrated false discovery rate (FDR) analysis function [24], [25]. The data were searched against the UniProtKB/Swiss-Prot database (downloaded in April 2011). The samples were described using the following parameters: sample type - iTRAQ 4plex (peptide labeled); Cys alkylation – methyl methanethiosulfonate; digestion - trypsin; special factors - no selection; species - *Homo sapiens*. The processing was specified as follows: quantitate - on; bias correction - on; ID focus - biological modifications; search effort - thorough; detected protein threshold - 0.05 (10.0%). Due to the possible protein and peptide ambiguity in the analysis of shotgun proteomic data, the Pro Group algorithm reported detected protein groups. Therefore, in instances where spectra or peptides can be assigned to more than one protein, ProteinPilot lists the alternative possibilities under the selected protein identity. For FDR determination, the software automatically searched data against concatenated database by *in silico* on-the-fly reversal for decoy sequences. Only proteins at 5% FDR were used for further analysis of the amniotic fluid data. Intensities of iTRAQ reporter ions were corrected using isotope correction factors supplied with the iTRAQ kit.

Only proteins with significantly altered abundance (*p*<0.01) in both replicates were considered for selection of biomarker candidates for verification and subsequent validation. Proteins were sorted based on the average iTRAQ quantitative change calculated from both replicates.

### Amniotic Fluid Cathelicidin ELISA Experiments

The concentration of cathelicidin LL-37 active form was determined in amniotic fluid using a commercial ELISA kit (Hycult Biotech, Uden, The Netherlands) in both exploratory and replication cohorts. The limit of detection of the kit was 0.14 ng/ml. Samples were diluted 1∶4 using phosphate buffer saline. Absorbance values were read at 450 nm using Multiskan RC ELISA reader (Thermo Fisher Scientific, Waltham, MA).

### Statistical Analyses of ELISA Data from Validation and Replication Cohorts

The demographic and clinical characteristics were compared using unpaired *t*-tests for continuous variables (presented as the mean ± SD) or the Mann-Whitney U test for nonparametric variables (presented as the median along with the range). Categorical variables were compared using the Fisher exact test and are presented as number (%). The normality of the data was tested using the D’Agostino and Pearson omnibus normality test and the Shapiro-Wilk test. Because concentrations of cathelicidin were not normally distributed, the nonparametric Mann-Whitney U test was used for analyses and data are presented as median [interquartile range (IQR)]. Receiver-operator characteristic (ROC) curves were constructed to determine the predictive value of cathelicidin for the presence of both MIAC and HCA. Cut-off point of amniotic fluid cathelicidin was chosen based on the maximum likelihood ratio (LR) calculated from exploratory cohort cathelicidin levels. Differences were considered statistically significant at *p*<0.05. All *p*-values were obtained from two-sided tests. All statistical analyses were performed using GraphPad Prism 5.03 for Mac OS X (GraphPad Software, La Jolla, CA), SPSS 19.0 statistical package for Mac OS X (SPSS Inc., Chicago, IL), and PASS 11 (NCSS, Kaysville, UT).

## Results

### Exploratory Phase of the Study

#### Demographic and clinical characteristics of the exploratory cohort

For the initial exploratory phase of the study we employed 19 amniotic fluid samples in each group to be compared. Table 1 presents the demographic and clinical characteristics of both women and newborns according to the presence and the absence of MIAC and HCA. All women were self-reported as Caucasians.

**Table 1:** Demographic and clinical characteristics of women and newborns involved in the exploratory cohort.

	Women with the absence of both MIAC and HCA (n = 19)	Women with the presence of both MIAC and HCA (n = 19)	*p*-value
**Maternal age (years)**	28.1±4.3	29.5±6.6	0.46
**Nulliparous**	11 (58%)	11 (58%)	1.00
**Smoking in pregnancy**	2 (11%)	6 (32%)	0.23
**Prepregnancy body mass index**	22.0 (17.6–33.2)	19.9 (17.0–33.0)	0.12
**PPROM to delivery interval (hours)**	6 (2–16)	8 (1–16)	0.50
**Gestational age at sampling (days)**	32+0 (24+1−34+5)	30+5 (25+2−34+5)	0.13
**Gestational age at delivery (days)**	32+0 (24+1−34+6)	31+0 (25+4−34+5)	0.23
**Birth weight (grams)**	1782±529	1572±401	0.18
**Apgar score <7 in 5 minutes**	1 (5%)	1 (5%)	1.00
**Apgar score <7 in 10 minutes**	0 (0%)	1 (5%)	1.00

Continuous variables were compared using parametric t-test (presented as mean ± SD) or a nonparametric Mann-Whitney U test [presented as median (range)]. Categorical variables were compared using Fisher exact test and presented as number (%). Abbreviations: MIAC  =  microbial invasion of the amniotic cavity; HCA  =  histological chorioamnionitis; PPROM  =  preterm prelabor rupture of membranes.

#### Exploratory proteomic analysis

**Figure 1:**
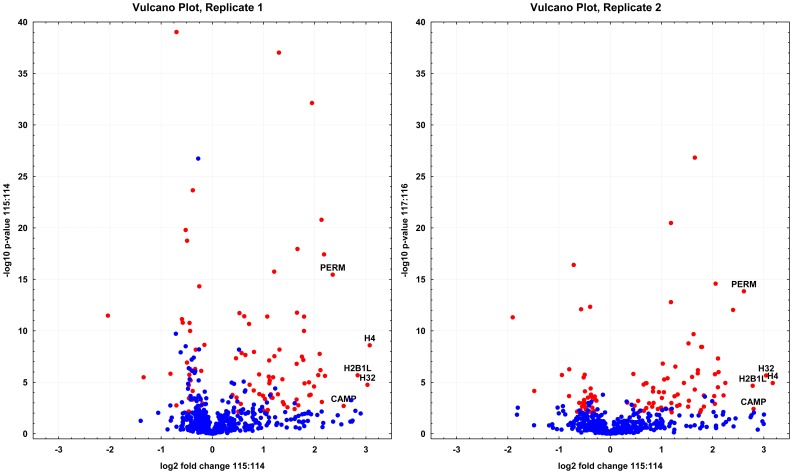
Volcano plots constructed from iTRAQ quantification data. The volcano plots show how much and how significantly proteins identified in the exploratory phase of the study using representative pooled samples were altered due to the presence of both MIAC and HCA. The top five proteins with the most profound change are highlighted by their abbreviated names (H4 - histone H4; H32 - histone H3.2; H2B1L - histone H2B type 1-L; CAMP – cathelicidine; PERM – myeloperoxidase). Proteins that were found significantly changed (*p*<0.01) in both replicates are coded in red.

The exploratory proteomic study of pooled amniotic fluid samples obtained from the exploratory cohort patients involved removal of 14 ballast proteins, peptide fractionation based on the presence of cysteine residues, initial separation on reversed-phase in basic conditions, and eventually reversed-phase HPLC-MALDI-TOF/TOF analysis (Fig. S1). This multidimensional nature of the study led to the recording of 20.382 MS/MS spectra, identifying 9.422 distinct peptides at a maximum of 5% FDR [25]. Based on these peptides, 851 amniotic fluid proteins were successfully identified (5% FDR). Of these, 99 proteins were significantly (*p*≤0.01) altered in both replicates (see Fig. 1 and Table S1 and S2). Three distinct histone proteins (P62805; Q71DI3; Q99880) showed the highest concentration change, followed by cathelicidin (P49913) and myeloperoxidase (P05164). These proteins thus represent five biomarker candidates with the most promising diagnostic potential for identifying MIAC leading to HCA. The multidimensional exploratory analysis led to the detection of proteins down to a few nanograms *per* ml concentration as implied from the cathelicidin levels measured by ELISA in subsequent steps.

#### Verification of the exploratory results concerning amniotic fluid cathelicidin levels

**Figure 2:**
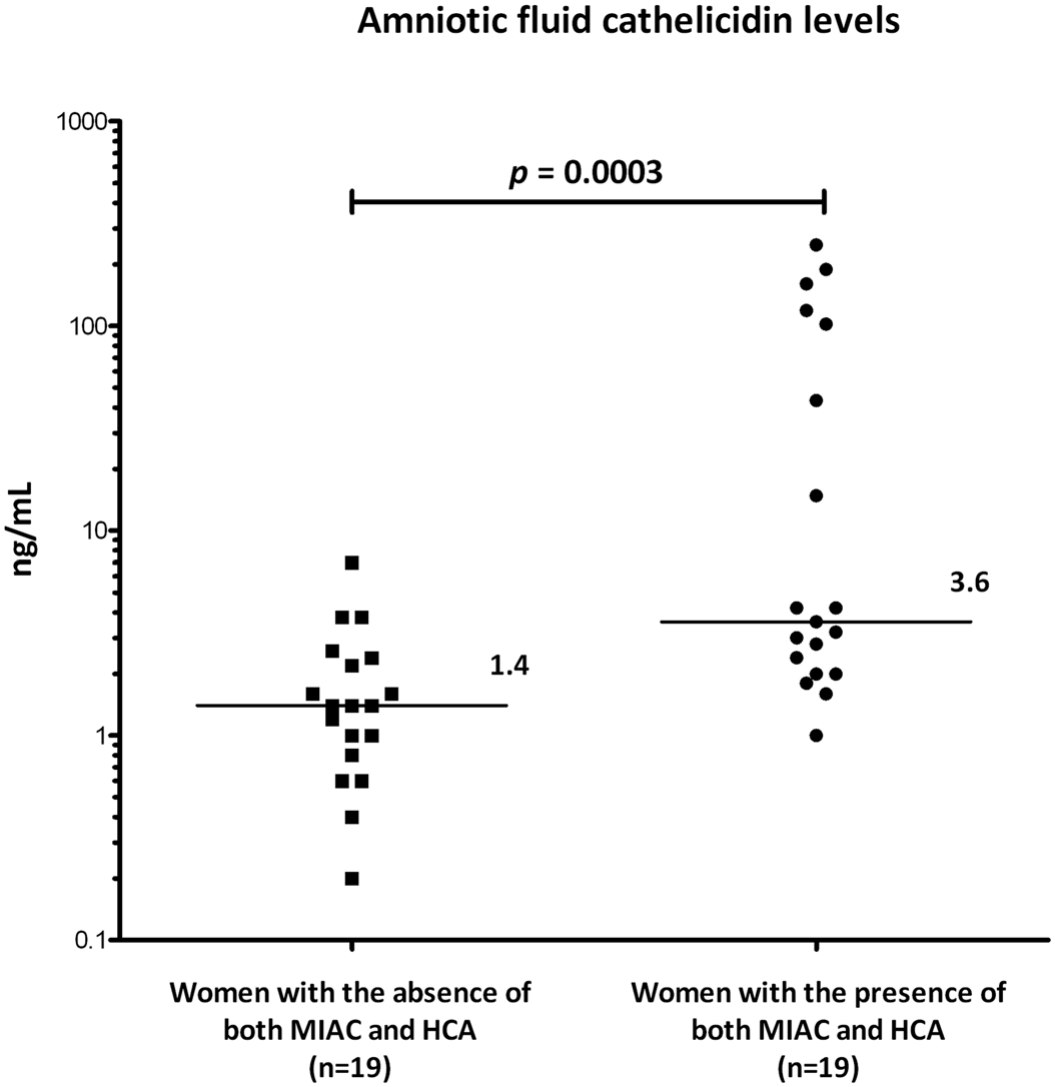
Verification of amniotic fluid cathelicidin levels. In concordance with the iTRAQ findings, women with confirmed MIAC and HCA enrolled in the exploratory phase had significantly higher amniotic fluid cathelicidin concentration than women in whom both conditions were ruled out. Cathelicidin levels were measured using a commercial ELISA kit. Values were evaluated using nonparametric Mann-Whitney U test. Graph represents individual values, horizontal bars indicate median values. Abbreviations: MIAC  =  microbial invasion of the amniotic cavity; HCA  =  histological chorioamnionitis.

To verify our exploratory proteomic data we used ELISA to assess cathelicidin levels in both groups of the exploratory cohort (Fig. 2). Exploratory cohort patients with the presence of both MIAC and HCA had higher amniotic fluid cathelicidin levels than women without both MIAC and HCA (the presence of both MIAC and HCA: median 3.6 ng/ml, IQR 2.0-102.2; with the absence of both MIAC and HCA: median 1.4 ng/ml, IQR 0.8–2.4; *p* = 0.0003).

### Validation Phase of the Study

#### Demographic and clinical characteristics of the replication cohort

An independent replication cohort was employed to further validate the verified findings regarding cathelicidin levels. To reach statistical power of 80% (α = 0.01), the size of the replication cohort was calculated and required at least 38 women in each group. Table 2 presents the demographic and clinical characteristics of the women and newborns with respect to the presence and absence of MIAC and HCA. Women with MIAC and HCA had lower gestational age at sampling, lower gestational age at delivery, and lower birth weight. Higher rates of MIAC, HCA, and funisitis were found in those with MIAC and HCA. All women were self-reported as Caucasians.

**Table 2:** Demographic and clinical characteristics of the women and newborns involved in the replication cohort.

	The other women (n = 63)	Women with the presence of both MIAC and HCA (n = 40)	*p-*value
**Maternal age (years)**	30.9±5.0	30.8±4.9	0.94
**Nulliparous**	32 (80%)	11 (28%)	0.03
**Smoking in pregnancy**	9 (14%)	11 (28%)	0.64
**Prepregnancy body mass index**	22.1 (17.5–36.7)	22.5 (17.0–35.7)	0.79
**Gestational age at sampling**	34+0 (24+0–36+5)	30+3 (24+0 - 35+1)	<0.0001
**Gestational age at delivery**	34+0 (24+4–36+5)	30+6 (24+1–35+2)	<0.0001
**The presence of MIAC**	8 (13%)	40 (100%)	<0.0001
**PPROM to delivery interval (hours)**	7 (2–23)	8 (1–25)	0.63
**Birth weight (grams)**	2000±598	1470±588	<0.0001
**Apgar score <7 in 5 minutes**	3 (5%)	3 (8%)	0.68
**Apgar score <7 in 10 minutes**	2 (3%)	2 (5%)	0.64
**The presence of HCA**	31 (49%)	40 (100%)	<0.0001
**The presence of funisitis**	4 (6%)	16 (40%)	<0.0001
**Postpartum endomyometritis**	2 (3%)	1 (3%)	1.00

Continuous variables were compared using parametric t-test (presented as mean ± SD) or a nonparametric Mann-Whitney *U* test [presented as median (range)]. Categorical variables were compared using Fisher exact test and presented as number (%). Abbreviations are explained in the legend for Table 1.

#### Validation of amniotic fluid cathelicidin in the replication cohort

**Figure 3:**
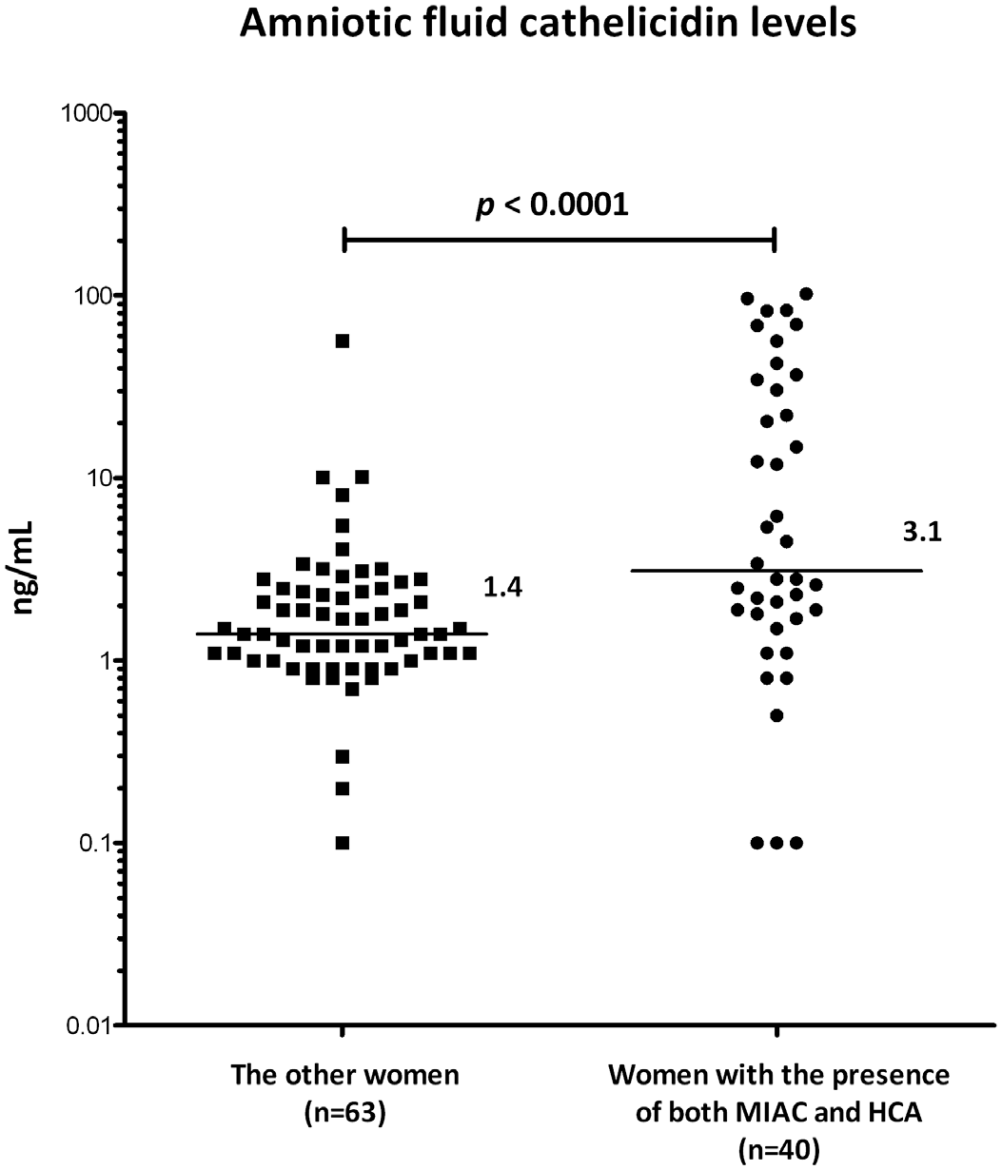
Validation of amniotic fluid cathelicidin levels on independent prospective replication cohort. Amniotic fluid cathelicidin levels in PPROM women with MIAC and HCA were compared with those in whom at least one condition was ruled out. The latter group involved women with neither MIAC nor HCA, without MIAC but with HCA, or with MIAC but without HCA. In line with the validation experiment, amniotic fluid cathelicidin concentrations were significantly higher in women with confirmed MIAC and HCA than in the other women. Cathelicidin levels were measured using a commercial ELISA kit. Values were evaluated using nonparametric Mann-Whitney U test. Graph represents individual values, horizontal bars indicate median values. Abbreviations are explained in the Fig. 2.

Women who had both MIAC and HCA had higher amniotic fluid cathelicidin levels than the rest of the women (the presence of both MIAC and HCA: median 3.1 ng/ml, IQR 17.0–34.6; other women: median 1.4 ng/ml, IQR 1.0–2.5; *p*<0.0001; Fig. 3).

### The Predictive Value of Cathelicidin for the Presence of both MIAC and HCA

To evaluate the diagnostic potential of cathelicidin in stratifying women with MIAC leading to HCA from the other women (women with neither MIAC nor HCA, without MIAC but with HCA, or with MIAC but without HCA), we calculated its predictive value. The amniotic fluid cathelicidin concentration of 4.0 ng/ml was found to be the best cut-off point based on LR (9.0) for identifying PPROM women with the presence of both MIAC and HCA in the exploratory cohort [sensitivity: 47%; specificity: 95%; odds ratio: 16.2; area under receiver-operating characteristic curve (AUC): 84% (Fig. S3)]. The same cut-off point of 4.0 ng/ml was subsequently tested on the validation cohort. A sensitivity of 48%, a specificity of 90%, an odds ratio of 11.6, an LR of 5.0, and an AUC of 71% were achieved for the prediction of women with MIAC and HCA in an independent prospective cohort (Fig. S3 and Table 3).

**Table 3:** Prediction potential of amniotic fluid cathelicidin cut-off level >4.0 ng/ml for the presence of both MIAC and HCA in PPROM pregnancies.

	Exploratory cohort	Replication cohort
**Area under curve (95% CI)**	0.84 (0.72–0.97)	0.71 (0.60–0.83)
***p*** **-value**	0.008	<0.0001
**Correctly predicted**	71%	74%
**Sensitivity (95% CI)**	47% (24–71)	48% (32–64)
**Specificity (95% CI)**	95% (74–100)	90% (80–96)
**Positive predictive value (95% CI)**	90% (56–100)	76% (55–91)
**Negative predictive value (95% CI)**	64% (44–81)	73% (62–83)
**Odds ratio (95% CI)**	16.2 (1.8–147.1)	11.6 (3.8–35.0)
**Likelihood ratio**	9.0 (1.3–64.3)	5.0 (2.2–11.4)

The differences among areas under curves are not statistically significant (*p*>0.05). Abbreviation: CI  =  confidence interval.

## Discussion

Advanced proteomic technologies enabled us to get an unbiased insight into the amniotic fluid proteome changes that occur due to the presence of MIAC leading to HCA. Cathelicidin was revealed among the top five proteins (Fig. 1 and Fig. S2), showing markedly and significantly different levels in the MIAC- and HCA-positive patient group compared with the group in which these findings were ruled out. We verified its differential abundance in the same cohort of women involved in the proteomic exploratory phase. Furthermore, we used a substantially larger and independent prospective replication cohort to validate cathelicidin potential for stratifying women with ongoing MIAC leading to HCA from the women in whom at least one of these conditions was ruled out.

To remain unbiased during the biomarker candidate selection from the initial proteomic data, we set the following criteria to filter the proteins of interest: FDR below 5%, change in abundance with *p*<0.01 in both replicates. The top five proteins in this list included three distinct histones, cathelicidin, and myeloperoxidase. The histones showed the most profound change in abundance. Although the role of this protein family during infection and inflammation is truly interesting, we were not able to find suitable ELISA assays for the verification of the whole group of these proteins from our analysis. However, we are in the process of implementing a technology that is capable of discriminating and quantifying individual histone proteins without the need for a specific antibody. Our proteomic findings regarding the histone proteins will thus be verified and validated in the near future.

A readily available ELISA kit for determining the levels of cathelicidin allowed us to focus our attention on this protein that, similarly to the detected histone proteins, showed a profound and significant increase in concentration due to the presence of MIAC and HCA (Fig. 1). Although cathelicidin was previously listed in proteomic projects focused on the identification of novel biomarkers of intraamniotic infection and inflammation, no verification and validation was undertaken to support the initial proteomic data and the diagnostic potential of cathelicidin [19], [20].

As mentioned above, myeloperoxidase also fulfilled our criteria for candidate selection. Although a suitable ELISA assay is available, the verification and validation of exploratory proteomic findings will be covered elsewhere. Thus, this work focused solely on the potential of cathelicidin to discriminate the group of PPROM women with MIAC leading to HCA from the women in whom both conditions were ruled out.

Several lines of evidence support a likely association of cathelicidin with MIAC leading to HCA. Cathelicidin is produced and released from epithelial cells, macrophages, and most importantly neutrophils upon stimulation by microorganisms. It was proved to be secreted in high amounts in tissues exposed to environmental microbes, particularly in those with squamous epithelia (mouth, tongue, cervix, vagina, esophagus, etc.) or in derived fluids [26]. It is expressed in a form of an inactive preprotein, which has to be proteolytically cleaved into antibacterial LL-37 peptide [27].

The crucial role of cathelicidin in fighting infection has been demonstrated both in patients [28], [29] and in experimental animal models, where cathelicidin-deficient mice were found to be more prone to infection [30], [31]. The antimicrobial effect was also confirmed experimentally in body fluids, including amniotic fluid or urine [30], [32]. Similar findings lead to elucidation of the supposed antimicrobial effect of vitamin D, which can activate cathelicidin production along with bacteria and viruses [33], [34]. The proposed mechanism of action is triggered by Toll-like receptor 2/1 activation, which leads to the production of 25-hydroxyvitamin D-1 α-hydroxylase, which in turn converts inactive 25-hydroxyvitamin D into active 1,25-dihydroxyvitamin D. This active form eventually binds to vitamin D receptor, a transcription factor that activates cathelicidin gene transcription [35].

The association between cathelicidin and vitamin D may be also regarded from another point of view. While vitamin D promotes antimicrobial agent production, it also has anti-inflammatory effects [36]. Even the “executing” component of the antimicrobial effect, cathelicidin, was shown to have anti-inflammatory influence [37]. Several studies have shown, that maternal vitamin D deficiency is associated with a range of pregnancy related morbidities and adverse neonatal outcome [38]–[42]. It can be speculated, that low levels of vitamin D may result in impaired production of antimicrobial peptides, which in turn could lead to reduced ability of facing microbial invasion. Given the fact that infection and/or inflammation are regarded as key components of causes leading to preterm birth, low vitamin D levels might be associated with increased risk of preterm labour [43].

Cathelicidin does not only participate in innate immunity as an antibacterial compound, but has also been shown to play an important role in modulating adaptive immunity [44]. It has also been shown to exhibit chemotactic activity and to attract neutrophils, monocytes, T-cells, and mast cells to the site of infection [45]–[47], where cathelicidin regulates inflammatory response and promotes tissue repair [48]–[50]. From the aforementioned it is very likely that cathelicidin amniotic fluid levels may indeed mirror ongoing MIAC leading to HCA.

The exact source of the elevated amniotic fluid cathelicidin level in our study remains unclear. We speculate, however, that granulocytes, neutrophils in particular, fetal, maternal, or both, are the predominant source of increased cathelicidin level in amniotic fluid. Our assumption is supported by the work of Klaffenbach et al., where the authors assessed antimicrobial peptides and protein production by placenta [51]. Although placental tissue is capable of producing a wide range of antimicrobial peptides, granulocytes were the key source of secreted proteins. Amniotic fluid is in close contact with the fetus; it surrounds the body surface, but is also swallowed and passes through multiple fetal compartments. Both the neonatal skin and the digestive tract have been described as being capable of producing cathelicidin [52]–[54]. Thus, the fetus may also contribute to the increased cathelicidin levels. Several other studies have suggested a role of cathelicidin in the urogenital and reproductive compartments [55], [56]. Zegels et al. analyzed human cervical-vaginal fluid using shotgun proteomics and detected cathelicidin along with other proteins and peptides with antimicrobial properties [56].

To the best of our knowledge, this is the first work to find an association of increased cathelicidin level with the presence of MIAC and HCA in amniotic fluid from PPROM patients, which was subsequently verified and then independently validated. Although previous proteomic studies already pointed to the presence of cathelicidin in amniotic fluid and suggested its association with occurring infectious and inflammatory processes, these findings were not verified by an independent, complementary approach nor were they validated in an independent patient cohort. The comparison of the diagnostic potential of cathelicidin in terms of identifying the infectious phenotype in PPROM patients with other potential biomarkers is rather complicated due to the phenotypic heterogeneity of the recruited cohorts, the use of different definitions for intraamniotic infection and HCA, and the methods for the detection of bacteria in amniotic fluid.

The key and principal strength of our work resides in the translational aspect of the study. We successfully applied a wide range of proteomic methods into obstetrics and gynecology focused research and showed that proteomics is capable of providing truly interesting results in the quest for novel biomarkers. Many other projects aimed at biomarker discovery unfortunately do not get past the exploratory proteomic phase, and thus, the published results are rather of limited significance. Keeping this in mind, we sought to go beyond this shortcoming and conducted a verification of our exploratory phase findings. Based on the obtained results, we went even further and successfully validated the findings in a larger, independent replication cohort. The likelihood ratios of 9.0 and 5.0 for the prediction of MIAC leading to HCA in PPROM pregnancies suggest that amniotic fluid cathelicidin is a valuable marker from a clinical point of view.

## Supporting Information

Figure S1 **The workflow of the exploratory proteomic analysis.** From both groups of exploratory phase samples two pooled representative replicates were prepared. After immunoaffinity depletion, flow-through proteins were digested with trypsin, and peptides were processed according to the CysTRAQ protocol. Peptides from both cysteinyl and non-cysteinyl fractions were eventually analyzed using two-dimensional HPLC separation and MALDI-TOF/TOF.
(TIF)

Figure S2 **iTRAQ proteomic data associated with cathelicidin (P49913).** For each identified cathelicidin peptide, its sequence, iTRAQ ratio for both replicates, segment of MS/MS spectrum with iTRAQ reporter ions, and whole-range MS/MS spectrum are shown. Representative pooled replicates of amniotic fluid samples from PPROM women in whom MIAC and HCA were ruled out were labeled by iTRAQ tags 114 and 116, while iTRAQ tags 115 and 117 were used for representative pooled replicates associated with the presence of both MIAC and HCA.
(TIF)

Figure S3 **Receiver operating characteristic (ROC) curves.** ROC curve is shown for amniotic fluid cathelicidin measured using ELISA in the exploratory cohort (A) and replication cohort (B) for the identification of PPROM women with both, MIAC and HCA.
(TIF)

Table S1 **Proteins identified in the exploratory proteomic phase of the study.** All identified proteins were exported from the ProteinPilot software and listed in spreadsheet **Raw Protein Data**, including all characteristics related to their identification and quantification. From these proteins, only those identified with FDR below 5% are presented in spreadsheet **Proteins below FDR 5%.** In spreadsheet **Protein Groups below FDR 5%** only names of detected protein groups were kept. Proteins found to be significantly changed in the exploratory phase are listed in spreadsheet **Protein Groups (** ***p*** **<0.01).** Here, proteins are sorted according to the absolute extremity of the change.
(XLSX)

Table S2 **Peptides identified in the exploratory proteomic phase of the study.** All identified peptides were exported from the ProteinPilot software and listed in spreadsheet **Raw Peptide Data**.
(XLSX)
