# Supplementary figures and images for: Amniotic Fluid Cathelicidin in PPROM Pregnancies: From Proteomic Discovery to Assessing Its Potential in Inflammatory Complications Diagnosis
Source: PLoS One. 2012 Jul 18;7(7):e41164. doi: 10.1371/journal.pone.0041164 (PMC3399859; doi:10.1371/journal.pone.0041164)

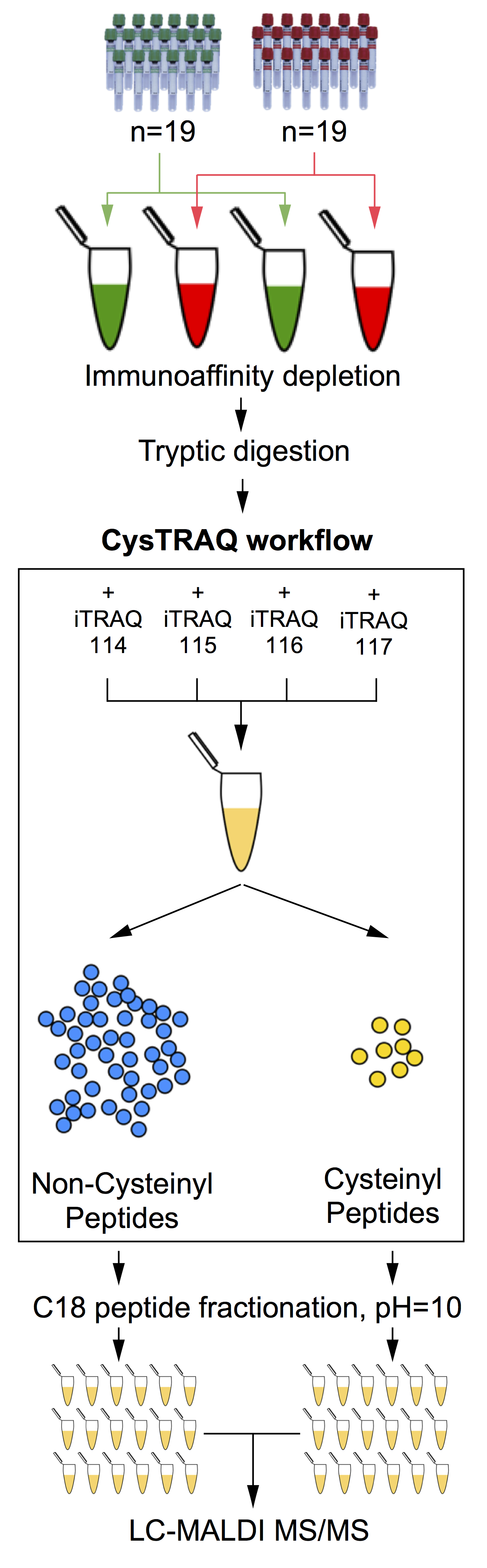

Supplement: Figure S1 — The workflow of the exploratory proteomic analysis. From both groups of exploratory phase samples two pooled representative replicates were prepared. After immunoaffinity depletion, flow-through proteins were digested with trypsin, and peptides were processed according to the CysTRAQ protocol. Peptides from both cysteinyl and non-cysteinyl fractions were eventually analyzed using two-dimensional HPLC separation and MALDI-TOF/TOF. (TIF) [file pone.0041164.s001.tif]

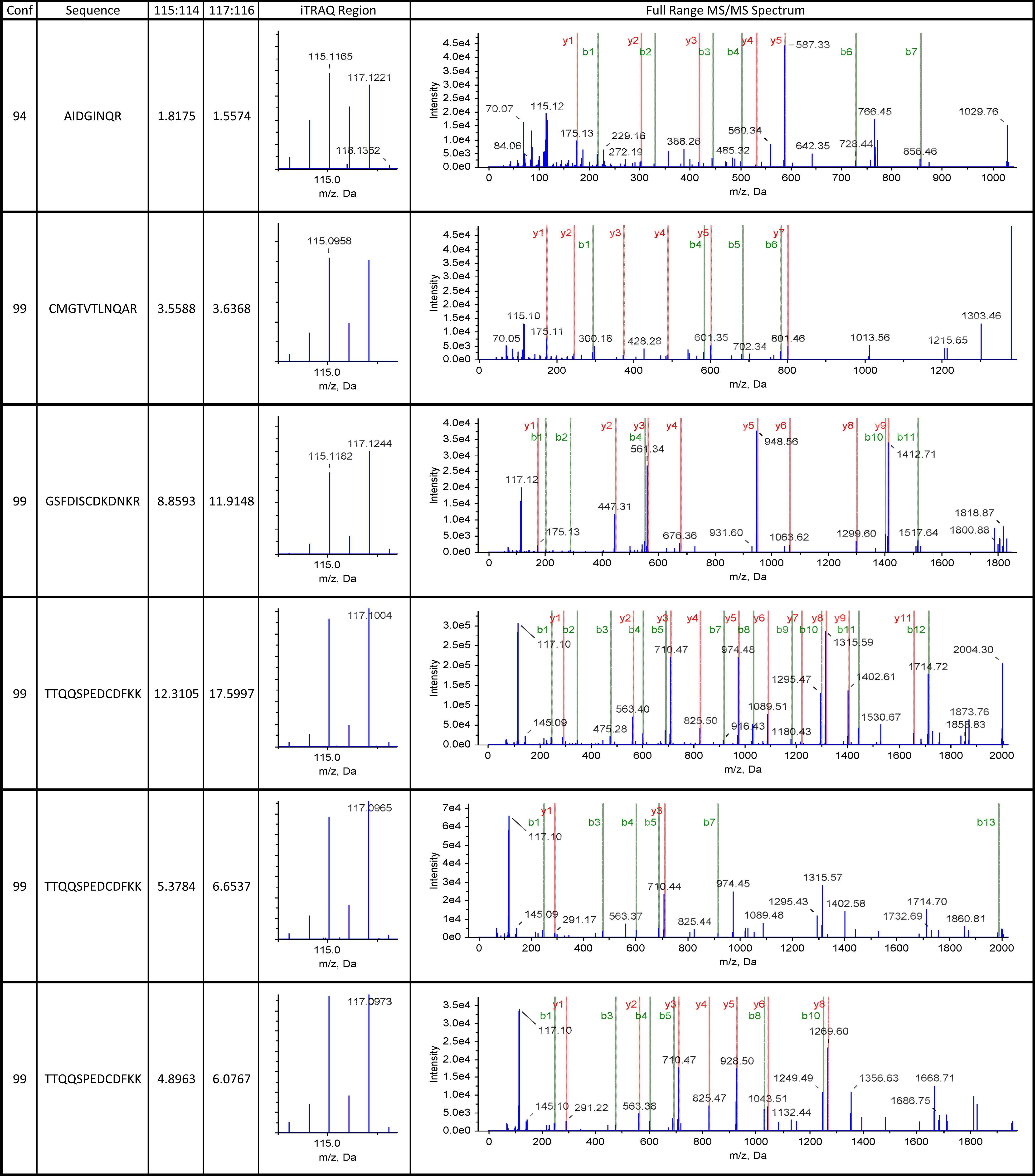

Supplement: Figure S2 — iTRAQ proteomic data associated with cathelicidin (P49913). For each identified cathelicidin peptide, its sequence, iTRAQ ratio for both replicates, segment of MS/MS spectrum with iTRAQ reporter ions, and whole-range MS/MS spectrum are shown. Representative pooled replicates of amniotic fluid samples from PPROM women in whom MIAC and HCA were ruled out were labeled by iTRAQ tags 114 and 116, while iTRAQ tags 115 and 117 were used for representative pooled replicates associated with the presence of both MIAC and HCA. (TIF) [file pone.0041164.s002.tif]

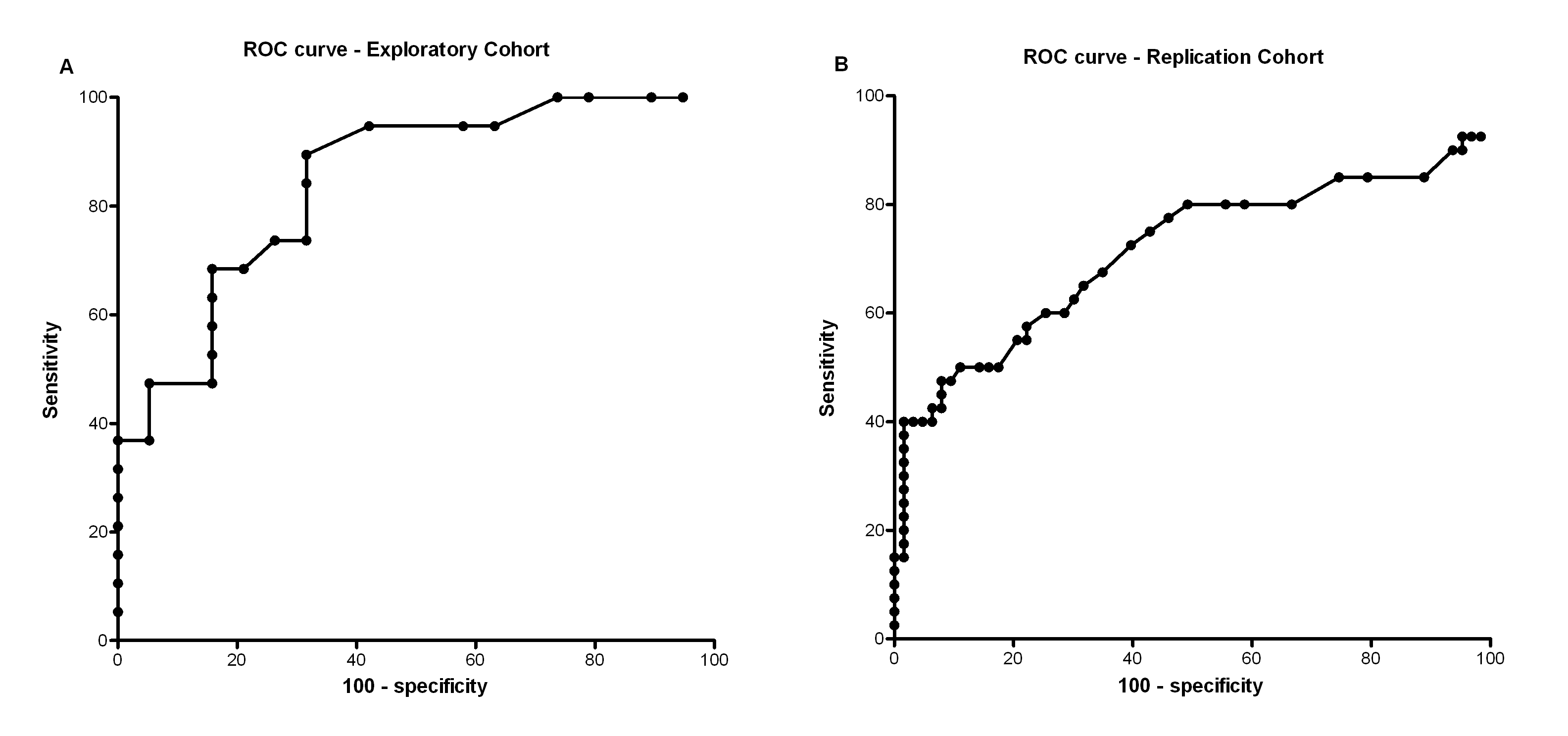

Supplement: Figure S3 — Receiver operating characteristic (ROC) curves. ROC curve is shown for amniotic fluid cathelicidin measured using ELISA in the exploratory cohort (A) and replication cohort (B) for the identification of PPROM women with both, MIAC and HCA. (TIF) [file pone.0041164.s003.tif]
